# Effect and mechanism of graphene structured palladized zero-valent iron nanocomposite (nZVI-Pd/NG) for water denitration

**DOI:** 10.1038/s41598-020-66725-z

**Published:** 2020-06-18

**Authors:** Xiangfeng Huang, Feifan Zhang, Kaiming Peng, Jia Liu, Lijun Lu, Shiyang Li

**Affiliations:** College of Environmental Science and Engineering, State Key Laboratory of Pollution Control and Resource Reuse, Ministry of Education Key Laboratory of Yangtze River Water Environment, Shanghai Institute of Pollution Control and Ecological Security, Tongji University, Shanghai, 200092 China

**Keywords:** Environmental sciences, Environmental chemistry, Environmental impact

## Abstract

Nitrate reduction by zero-valent iron-based materials has been extensively studied. However, the aggregation of nanoparticles and the preference for unfavored ammonia products limit the application of this technology. To overcome this issue, this study introduced a novel synthesized nanoscale palladized zero-valent iron graphene composite (nZVI-Pd/NG) and explored its nitrate reduction efficiency. A nitrate removal rate of 97.0% was achieved after 120 min of reaction for an initial nitrate concentration of 100 mg N/L. The nitrogen gas selectivity was enhanced from 0.4% to 15.6% at the end point compared to nanoscale zero-valent iron (nZVI) particles under the same conditions. Further analyses revealed that zero-valent metal nanoparticles spread uniformly on the graphene surface, with a thin layer of iron (hydr)oxides dominated by magnetite. The nZVI-Pd/NG exhibited good catalytic activity with the associated activation energy of 17.6 kJ/mol being significantly lower than that with nZVI (42.8 kJ/mol). The acidic condition promoted a higher nZVI utilization rate, with the excess dosage of nZVI-Pd/NG ensuring a high nitrate removal rate for a wide pH range. This study demonstrates an improvement in nitrate reduction efficiency in a nZVI system by combining the exceptional properties of graphene and palladium.

## Introduction

Nitrate is a major environmental pollutant in surface and ground water systems^1,2^. Nitrate overload in water causes eutrophication and poses a threat on human health when consumed^3–5^. Several onsite and offsite methods, including biological denitrification^6,7^, reverse osmosis^8^, adsorption^9,10^, chemical reduction^3,11^, and electrolysis^12,13^, serve for nitrate removal. Among these treatment methods, chemical reduction is considered among the most promising due to its high efficiency and stability^14,15^.

Recently, the zero-valent iron (ZVI) has been intensively studied as a reductive material due to its non-toxicity, low cost, and wide availability. Previous studies demonstrate that nitrate is significantly reduced by metallic iron under anoxic and anaerobic conditions^2,16^. The surface area of iron used for denitration is vital in accelerating the reaction rate^17^. Therefore, nanoscale zero-valent iron (nZVI) with high surface area and nanoscale particle size are utilized for nitrate removal. The reaction rate of the nZVI is largely improved compared with the microscale ZVI, with the reaction time dropping from several days to 1-2 hours^16,18^. However, in practice, the agglomeration and passivation of nZVI particles reduce their effectiveness. The attraction between nZVI particles causes aggregation into microscale particles, thereby reducing their mobility and lowering the effective surface area^19^. The microscale particles formed are considered to behave like the microscale ZVI^20^. It has also been reported in many studies that as reaction proceeds, iron corrosion products (such as maghemite, lepidocrocite, magnetite, akaganeite, goethite) may cover the core-shell structure of nZVI, preventing subsequent reactions^21,22^. Furthermore, unfavored ammonia emerges as the major reduction product, without a consensus method for enhancing the selectivity of nitrogen gas^23,24^.

To overcome these problems, experiments involving modified materials attracted significant attention from researchers. Firstly, doping noble metals like Pd, Pt, and Rh is indicated to enhance the reducibility of nZVI dramatically^25,26^. Since noble metals are catalysts for hydrogen formation, the activation and sequential formation of atomic hydrogen in solution could cause hydrogenation of pollutants, thus, facilitating pollutant transformation^27,28^. Embedded noble metals also show potential for protecting nZVI from passivation^29^. Moreover, regarding catalytic removal of nitrate, noble metals have been intensively studied for their selectivity towards end products^14,30^. Generally, Pd-based catalysts exhibit higher catalytic activity and N_2_ selectivity than other noble metals^19,31^. However, the above-mentioned noble metals are often employed as an essential part of bimetals under a hydrogen feeding atmosphere^5,25,27^. The catalytic effect of Pd for denitration with nZVI principally involved in the reduction process remains poorly investigated.

Moreover, supporting materials, including zeolite^32,33^, clay^34,35^, resin^36,37^ and activated carbon^38,39^, are introduced to hold the nanoparticles for effective stabilization and distribution. The deposition of nZVI on supports improves denitration performance by relieving their self-agglomeration and offering a high surface area for mass transfer^33^. Compared with these traditional supporting materials, graphene can improve the reaction performance due to its high surface area and unique interlayer space, as well as good electrical, mechanical, optical, and thermal properties suitable for heterogeneous reactions^40–43^. Graphene as a supporting material can significantly improve the dispersibility of the nanoparticles and assist in eliminating nanoparticle agglomeration during the reaction with different contaminants^44,45^. Moreover, graphene-based composite photocatalysts (e.g., 3D graphene aerogels and hydrogels^46,47^) have been proven beneficial for the degradation of environmental pollutants due to their excellent optical transmittance^48,49^. Studies also revealed its capacity to provide sites for the precipitation of iron (hydr)oxides during the reaction process^22^. More importantly, the conjugate structure of graphene enhances the adsorption capacity of reactant molecules, with the high electron mobility facilitating the transfer of electrons in the catalytic reaction, thereby improving the catalytic activity of nZVI particles^29^.

Reports on the support of nZVI on graphene are scant for aqueous nitrate reduction, with physical mixing of nZVI and graphene commonly applied relative to chemical impregnation^23,50^. To the best of our knowledge, bimetal nanoparticles (i.e., zero-valent iron and palladium) embedded in graphene for nitrate removal have not yet been reported, with the synergistic effect of graphene and a catalyst on nitrate reduction also lacking comprehensive studies.

This study introduces a novel synthesized nanoscale palladized zero-valent iron graphene (nZVI-Pd/NG) composite, and its nitrate removal efficiency was investigated through batch experiments. Several characterization methods were applied to examine the structural advantage of the nZVI-Pd/NG, with its catalytic activity further evaluated through activation energy determinations. The surface passivation process was also studied throughout the reaction process, and the effect of graphene, along with palladium was substantially explored.

## Results and Discussion

### Characteristics of fresh nZVI-Pd/NG

In this study, the properties of the freshly prepared nZVI-Pd/NG were investigated through BET, SEM, EDS, XRD, and XPS analyses, with fresh nZVI and graphene also characterized for comparison.

The textural properties of nZVI and nZVI-Pd/NG were tested using BET analyses, and the nitrogen adsorption isotherm, and pore size distribution curve of the nZVI-Pd/NG are shown in Fig S1. The mesoporous structure is characterized by the type IV isotherm showing the range of diameters for pores in nZVI-Pd/NG^51^. The BET surface area, average pore size, and pore volume results are presented in Table S1. After the nZVI particles were supported on graphene, the surface area increased from 11.05 to 28.07 m^2^/g, along with a significant rise in pore volume. Additionally, the embedding of Fe and Pd in graphene was inferred from the decrease in surface area and pore volume of the graphene.

**Figure 1:**
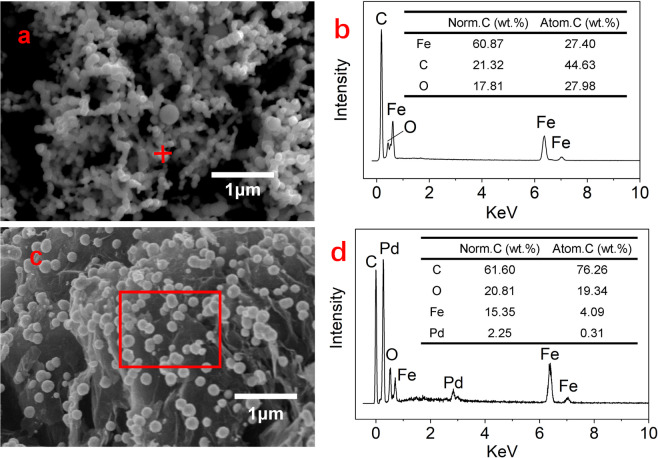
SEM graph and EDS intensity graph of (**a**,**b**) nZVI; (**c**,**d**) nZVI-Pd/NG.

The morphological structures of the prepared nZVI and nZVI-Pd/NG were investigated using SEM (Fig. 1a,c). Obviously, Fig. 1c reveals that the nanoparticles dispersed individually on the graphene surface without agglomeration. The diameter of the prepared nZVI determined from the Zetasizer was 1.4 μm, while the particle sizes of the nZVI doped on graphene measured by SEM ranged from 100–200 nm. From the perspective of morphology and texture, the nZVI-Pd/NG exhibits a good potential for promoting the nitrate reduction process, since a higher surface area and smaller particle size are conducive to the mass transfer process, and consequently enhance the reaction efficiency.

Figure 1b,d show the EDS intensity graphs of nZVI and nZVI-Pd/NG at a certain point or area. The O peak occurred in both graphs, suggesting the surface oxidation of iron. The intensity data also depicted a weight ratio of Fe: Pd of 6.82:1, being like the preconceived ratio of 3:0.5, and the good distribution of nZVI and Pd are illustrated by the EDS mapping graphs in Fig S2.

**Figure 2:**
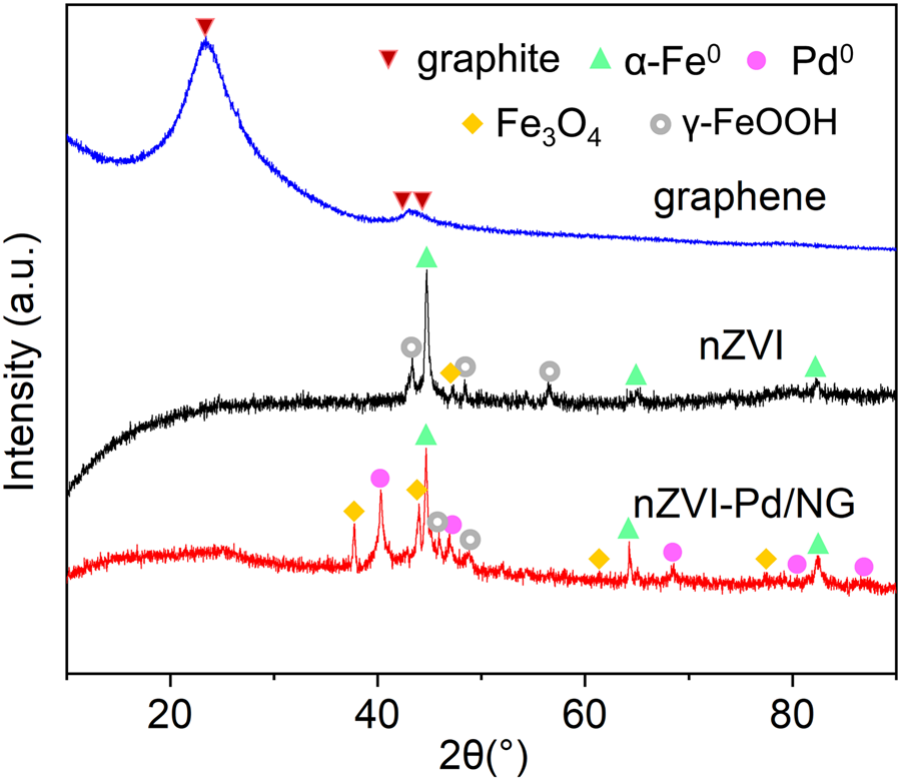
XRD patterns of fresh graphene, freshly synthesized nZVI, and nZVI-Pd/NG.

The crystal structures of the nanoparticles were further investigated by XRD analyses. The XRD pattern of graphene, nZVI, and nZVI-Pd/NG are displayed in Fig. 2. The diffraction peaks at 2θ angles of 23.4°, 43.0°, and 44.3° accord with peaks of native graphite (JCPDS 75-1621)^23^. The XRD patterns of nZVI and nZVI-Pd/NG exhibit peaks at 2θ of 44.6°, 64.8°, and 82.2°, that were assigned to the typical characteristics of α-Fe^0^ (JCPDS 06-0696) representing the (1 1 0), (2 0 0), and (2 1 1) planes in the crystal phase, respectively^32^. Moreover, the presence of different iron oxides was also revealed, with magnetite (Fe_3_O_4_, JCPDS 72-2303) and lepidocrocite (γ-FeOOH, JCPDS 74-1877) present in nZVI and nZVI-Pd/NG. Furthermore, the diffraction peaks at 2θ of 40.3°, 46.9°, 68.3°, 81.5°, and 86.6° in the XRD pattern of nZVI-Pd/NG perfectly match the corresponding peaks of palladium (JCPDS 88-2335), highlighting the synthesis of zero-valent palladium.

XPS measurements were also performed to investigate the composition and the chemical state of nZVI-Pd/NG. Fig S3 shows the XPS survey spectrum revealing the presence of C, Pd, O, and Fe in the sample. The high resolution Pd 3d,Fe 2p,and C 1 s XPS spectra and their deconvolution reveal the species of Fe and Pd are Fe^2+^ and Fe^3+^ and Pd^0^ and Pd^2+^, respectively. Since the XPS is a typical surface analysis method revealing the chemical states of elements within several nanometers, zero-valent iron is hardly detected on the surface of nZVI-Pd/NG. The O spectra shows the existence of M-O and –OH, with the three peaks in the C spectrum depicting the typical bonding of graphene.

Based on the former results, the nanoscale zero-valent iron and palladium were synthesized and well-distributed on graphene, with minimal agglomeration. Furthermore, smaller particle sizes and higher surface areas were achieved after supporting the nZVI.

### Nitrate removal performance

The nitrate removal performance of nZVI-Pd/NG was investigated by comparing nZVI-Pd/NG with four other materials (i.e., nZVI/NG, nZVI, nZVI-Pd, and Pd/NG). Five materials were applied to identical nitrate systems and compared based on the nitrate removal rate and reaction rate.

**Figure 3:**
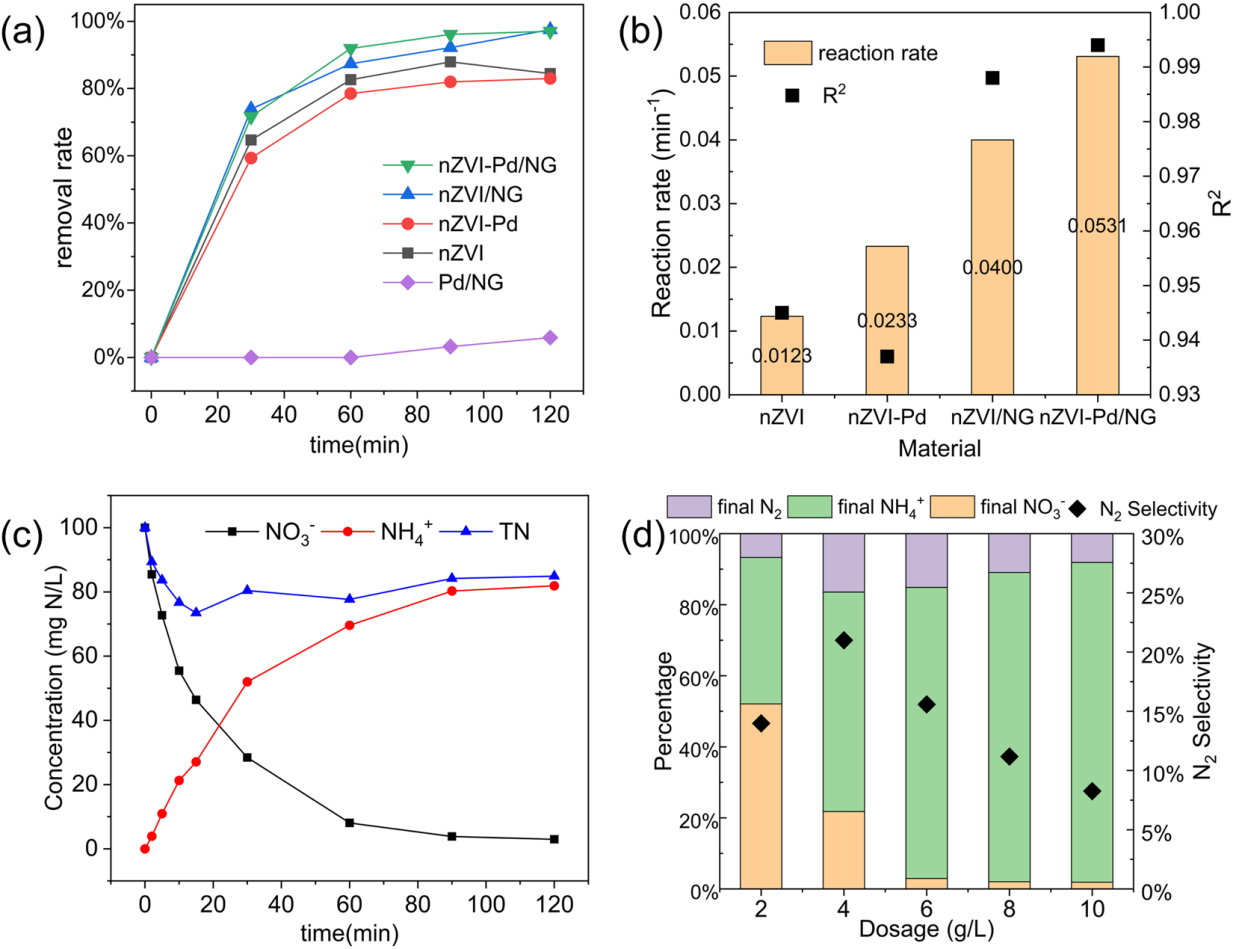
(**a**) Nitrate removal rate of nZVI, nZVI-Pd, nZVI/NG, Pd/NG, and nZVI-Pd/NG; (**b**) Reaction rates of nitrate reduction by different materials; (**c**) Nitrogen species during the reaction process in the nZVI-Pd/NG system; (**d**) Nitrogen species and nitrogen gas selectivity after 2 h of reaction at different nZVI-Pd/NG dosages. (Initial nitrate concentration = 100 mg N/L, pH = 7, temperature = 293 K, and dosage = 6.0 g/L).

The experiments were conducted at room temperature and neutral pH, with a different dosage of the five materials based on the equivalent dosage of 3.0 g/L nZVI or 0.5 g/L Pd. Samples were collected at intervals of 30, 60, 90, and 120 min for material comparison and the results are shown in Fig. 3a. The materials containing nZVI had a nitrate removal rate higher than 80% after 120 min of reaction, while Pd/NG barely showed any denitration ability, with neither reduction nor adsorption. This could be attributed to the decisive role of nZVI in the nitrate reduction process. Effective nitrate reduction was possible in the presence of zero-valent iron, while the Pd/NG The nZVI-Pd/NG and nZVI/NG produced similar nitrate removal efficiencies (97.0% and 97.5%, respectively) after 2 h of reaction, which is comparable to reported results from optimum conditions^14,16^, while nZVI and nZVI-Pd yielded 84.4% and 83.0% removal, respectively. Except for Pd/NG, all four materials showed similar trends in the nitrate removal rate, characterized by a quick transition within the first 30 minutes, with equilibrium attained after a 120-minute reaction, consistent with previous studies^38,52^. The results suggest that although thin layer iron oxides considered unfavorable for redox reactions were detected, our prepared catalysts adequately demonstrated their nitrate removal capability.

To further explore the kinetics of the nitrate reaction process, a pseudo-first kinetic model was applied to nZVI/NG, nZVI, nZVI-Pd, and nZVI-Pd/NG. The reaction process fits well with the pseudo first-order model (R^2^ > 0.93), enabling the calculation of the reaction rate of different materials (Fig. 3b). The nZVI-Pd/NG produced the highest reaction rate of 0.0531 min^−1^, that is quadruple that for nZVI under identical conditions, and displays superiority compared with materials reported in other studies (e.g., 5% Nano-Cu/Fe^53^, nZVI@MWCNTs^52^, nZVI@rGO^24^). The reaction rate of nZVI-Pd was promoted by the addition of Pd, however, it was still below that of nZVI/NG. According to previous studies, when the reactivity of nZVI is inhibited by self-aggregation, supporting the nZVI and improving its distribution is more effective than simply adding a catalyst, owing to the nano-size effect of the smaller aggregate size^19^. By comparing the reaction rate of the four materials, it is evident that the dispersion of nZVI by graphene and the catalytic effect of Pd enhance the denitration reactivity, while the support strengthens the nano-size effect.

The nitrate reduction process by nZVI-Pd/NG is specifically illustrated over time in Fig. 3c. The concentration of nitrate decreased continuously, reaching 2.98 mg N/L after 120 min of reaction, while the ammonium concentration increased simultaneously and ultimately reached 81.9 mg N/L. The loss of total nitrogen (TN) is attributed to conversion to nitrogen gas, with nitrite undetected throughout the reaction process in our study. Nitrite is widely regarded as an intermediate in nitrate reduction, and some studies report a small amount at the beginning of the reaction^52,53^. However, the occurrence is commonly associated with bimetal catalytic systems, where the characteristics of the noble metal determine the reaction rate from nitrite to the end products, as well as the ZVI systems that required days to achieve equilibrium^15,23^. The negligible amount of nitrite in our study is possibly due to the high reactivity of nZVI in nZVI-Pd/NG, with the produced nitrite rapidly converted to end products.

Since the reduction of nitrate relies on the zero-valent iron as an electron donor, the dosage of the reducing material is an essential factor in the reaction. Therefore, experiments were conducted at different nZVI-Pd/NG dosage levels, with the final species of nitrogen after 2 h of reaction depicted in Fig. 3d. When 2.0 g/L nZVI-Pd/NG was added to the solution, 47.9% of nitrate was transferred to other species. The removal rate of nitrate was greatly enhanced with an increasing mass of nZVI-Pd/NG, reaching 97.0% for 6.0 g/L nZVI-Pd/NG. The final nitrate concentration showed no significant change with additional nZVI-Pd/NG, consistent with previous reports^50,54^. Incremental nZVI-Pd/NG dosage at certain nitrate solution concentrations implies that more active sites of iron nanoparticles are provided for the nitrate ion, thereby accelerating the collision of ions in the initial reduction process and promoting thorough denitration^23^. The results of our study suggests that 6.0 g/L nZVI-Pd/NG provide adequate reactive sites for the reduction of 100 mg N/L of nitrate.

The selectivity towards different end products and the final pH of the solution varied with the range of complete nitrate removal. Nitrogen selectivity rose from 14.0% to 21.0% when the dosage increased from 2.0 to 4.0 g/L. A downward trend of nitrogen production was obtained with the incremental dosage of nZVI-Pd/NG after 4.0 g/L. This behavior is attributed to the increase in Pd sites from an increased material loading. More hydrogen radicals are produced at higher Pd loadings, accelerating the reduction process from nitrate to nitrite, which was equally useful for the recombination of N-H and N-N. However, the excess amount of catalyst likely promoted the formation of N-H relative to N-N, thus, increasing the production of ammonium^5,35^. Thus, in our study, the nZVI-Pd/NG dosage of 6.0 g/L was adequate for the reduction of 100 mg N/L of nitrate, with a nitrate removal rate and nitrogen selectivity of 97.0% and 15.6%, respectively.

### Catalytic activity

**Figure 4:**
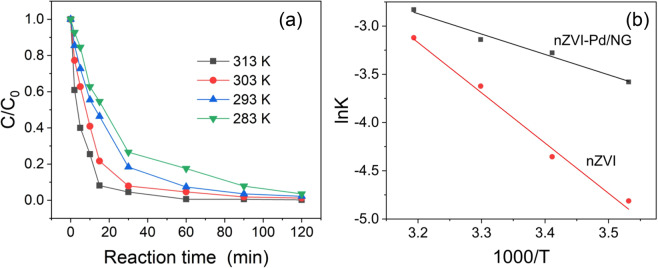
(**a**) Nitrate removal of nZVI-Pd/NG at different temperatures, dosage = 6.0 g/L, nitrate concentration = 100 mg N/L, pH = 7 and (**b**) Arrhenius plot for the estimation of the activation energy.

The catalytic activity of nZVI-Pd/NG was evaluated based on its nitrate removal efficiency at different temperatures. Temperatures ranging from 283 to 313 K were involved for nitrate removal experiments by nZVI and nZVI-Pd/NG. Samples were collected after 3, 5, 10, 15, 30, 60, 90, and 120 min of reaction, with the ratio of the remaining nitrate from nZVI-Pd/NG reduction calculated and depicted in Fig. 4a. The nitrate removal was 91.9% after 15 min at 313 K compared with only 45.4% at 283 K. The nitrate was gradually reduced as the reaction progressed, and after 90 minutes of reaction, the removal rate reached 92.2%, 96.5%, 98.2%, and 99.5% for the temperatures of 283 K, 293 K, 303 K, and 313 K, respectively. A higher reaction temperature probably increases the percentage of activated molecules and the collision probability between the nitrate ions and materials, producing better removal efficiencies with increasing temperature^29^. Contrarily, a decreasing temperature caused a smaller nitrate reduction, which is typical for an endothermic reaction, with a similar trend reported in nitrate reduction systems where zero-valent iron acted as the reductant^17,50,55^. According to these reports, the nitrate removal efficiency dropped from 85.6% (316 K) to 70.4% (298 K) by ZVINP/NG^50^, and similarly, only 60% of nitrate was removed by nZVI@rGO at 283 K after 60 min of reaction^24^. Compared with other studies, nZVI-Pd/NG shows superiority in reducing nitrate at low temperature.

The reaction kinetics of nZVI-Pd/NG and nZVI were further compared, with the corresponding reaction rates shown in Table S2. At each temperature, the reaction rate of nZVI-Pd/NG was 2–5 times higher than that of nZVI. The reaction rate of nZVI dropped to 0.0087 min^−1^ at 283 K, while nZVI-Pd/NG maintained a reaction rate of 0.0393 min^−1^. Clearly, low temperature impacts nZVI-Pd/NG less than nZVI.

The activation energy (E_a_) of the reaction was calculated by Arrhenius equation, with the slope of Fig. 4b yielding the value of E_a_. The activation energy is the potential energy that must be overcome for complete nitrate reduction and zero-valent iron oxidation^53^. During the process, nitrate diffusion, adsorption, chemical reaction, and products diffusion are all included, and the value of activation energy provides information on the limiting step for the reaction^50^. Diffusion requires less energy than a chemical reaction, and the value of the activation energy of a typical diffusion controlled reaction in water ranges from 10–20 kJ/mol^56^. In this study, nitrate reduction in batch experiments using nZVI and nZVI-Pd/NG produced E_as_ of 42.8 and 17.6 kJ/mol over the temperature of 283 K to 313 K. The value of E_a_ of nZVI was high enough to determine the reaction as an activation-controlled process, therefore, the degree of reaction control on the kinetics in the nZVI system was very important. The low apparent activation energy of nZVI-Pd/NG obtained confirms that the nitrate reduction by the new material follows a diffusion-controlled reaction^55^.

**Figure 5:**
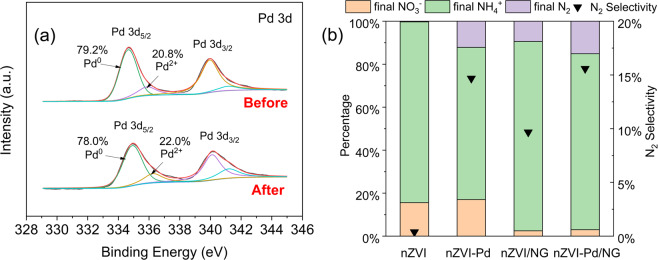
(**a**) XPS spectra of Pd on nZVI-Pd/NG before and after reaction and (**b**) nitrogen species after reaction and N_2_ selectivity by different materials.

The enhancement of the catalytic activity by nZVI-Pd/NG was further examined through the chemical state of palladium, with the XPS spectra of Pd before and after the reaction displayed in Fig. 5a. The two peaks at 334.93 and 340.14 eV represent Pd(0)^5^, and the peaks barely changed after the reaction. The constant oxidation state indicates that palladium did not directly participate in the NO_3_^-^ reduction but only acted as a catalyst for hydrogenation. Protons from water are reduced to atomic hydrogen and molecular hydrogen via iron corrosion^19^.

The catalytic effect of Pd was further assessed by comparing the end products in systems with different materials. The nitrogen species after 120 min of reaction and corresponding nitrogen selectivity are shown in Fig. 5b. By combining palladium with iron to form a bimetal, the nitrogen selectivity increased from 0.38% (nZVI) to 14.7% (nZVI-Pd). Similarly, the percentage of nitrogen gas generated increased from 9.7% to 15.6% when adding palladium to nZVI/NG. The two pairs of materials (i.e., nZVI and nZVI-Pd, nZVI/NG and nZVI-Pd/NG) had the same amount of nZVI and were similar in microstructure; the only difference was the embedded Pd. Thus, the catalytic effect of palladium on N_2_ generation could be confirmed. The palladium catalyzes nitrate reduction by the hydrodeoxygenation process, accelerating the cleavage of N-O bonds and sequentially forming N_2_ and NH_4_^+^^31^. Although the pathway for the end products are controversial, the catalyst structure and solution conditions are usually vital factors^57^. Some studies suggest that nitrogen formation is enhanced at high-coordinated metal sites (e.g., terrace sites), while ammonia formation is facilitated at low-coordinated metal sites (e.g., steps, edges, kinks)^58^. In our study, palladium was low in amount compared with nZVI, so that palladium was more likely to be deposited on iron particles separately or distributed directly on graphene. Isolated Pd favors separate NO_2_^−^ adsorption, promoting NH_3_ formation, nevertheless, Pd crystallites on graphene cause the generation of nitrogen and ammonia^31^.

The enhancement of N_2_ production by graphene could be deduced by comparing the results of nZVI with nZVI/NG, and nZVI-Pd with nZVI-Pd/NG, as shown in Fig. 5b. An increase of the N_2_ selectivity are indicated by increasing values from 0.38% (nZVI) to 9.7% (nZVI/NG), while nZVI-Pd and nZVI-Pd/NG showed similar results. It has been reported that the ratio of adsorbed nitrate ions to protons, described as N: H, could affect the reduction pathway of nitrate^14,59^. Higher N:H values favor N_2_ production, while lower values promote NH_4_^+^ formation. By introducing graphene to nZVI, the larger surface area greatly enhances nitrate adsorption, thereby increasing the N: H value, so that the generation of N_2_ was facilitated. The comparative N_2_ production by nZVI-Pd with nZVI-Pd/NG could be attributed to the catalytic effect of palladium when more H was generated, which, thus, limited further N_2_ production.

### Surface passivation and transformation

The freshly synthesized nZVI-Pd/NG was covered with iron (hydr)oxides, and that was believed inevitable due to the high reactivity of nZVI. However, the composition of iron (hydr)oxides and their further transformation likely impact the reaction process significantly.

During the study of nZVI evolution in an aqueous environment, the mixture of FeO, α-FeOOH, and/or β-FeOOH are present under anoxic conditions, whereas in oxic conditions, surface composites convert to magnetite/maghemite, and finally to lepidocrocite^21^. Therefore, the detected species of iron (hydr)oxides likely reflect the progress of iron corrosion. Some studies also examine the electron transfer ability, with lepidocrocite considered detrimental to electron transfer and mainly accounting for surface passivation, whereas magnetite facilitates the transfer of electrons from nZVI to the solid-liquid interface^60,61^. Comparing the XRD patterns of nZVI-Pd/NG and nZVI in Fig. 2, the peaks of nZVI-Pd/NG fitted better for magnetite than nZVI, and conversely, the pattern of nZVI fitted the peaks for lepidocrocite. Therefore, we propose that the oxidation layer of nZVI-Pd/NG is more conductive for electron transfer, so better iron utilization and higher reactivity are achievable.

The surface (hydr)oxides layer may dissolve and further precipitate, which was significant influenced by the aqueous pH. The acidic condition is beneficial to surface protonation and could weaken the metal oxygen bonds close to the surface central ions, and consequently facilitate the detachment of the metal species^62^. Moreover, nitrate reduction is a proton consuming process (as shown in the following equations), and as the reaction proceeds, the aqueous pH increases and stays alkaline, promoting iron precipitation^16^.

1 $$5{{\rm{Fe}}}^{0}+2{{\rm{NO}}}_{3}^{-}+12{{\rm{H}}}^{+}\to 6{{\rm{H}}}_{2}{\rm{O}}+5{{\rm{Fe}}}^{2+}+{{\rm{N}}}_{2}$$

2 $$4{{\rm{Fe}}}^{0}+{{\rm{NO}}}_{3}^{-}+10{{\rm{H}}}^{+}\to 3{{\rm{H}}}_{2}{\rm{O}}+4{{\rm{Fe}}}^{2+}+{{\rm{NH}}}_{4}^{+}$$

**Figure 6:**
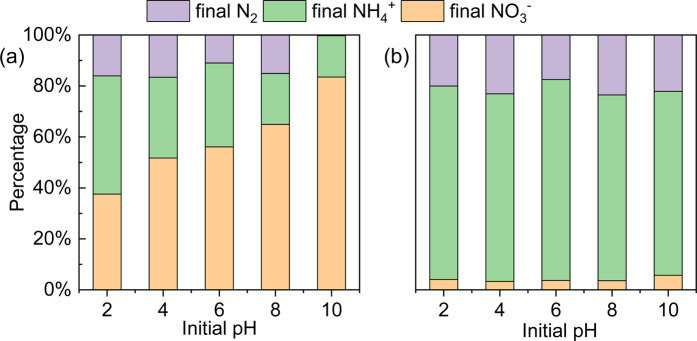
Effect of initial pH on nitrogen species after 120 min reaction (**a**) dosage = 2.0 g/L, (**b**) dosage = 6.0 g/L (nitrate concentration = 100 mg N/L, temperature = 293 K).

The impact of the initial pH on the nitrate reduction process and transformation of the surface passivation layer were studied at two dosage levels (2.0 g/L and 6.0 g/L). When 2.0 g/L nZVI-Pd/NG was added into the denitration system (Fig. 6a), the optimum nitrate removal was obtained at a solution pH of 2. As the pH value increased, the percent nitrate removal decreased correspondingly, in accordance with former studies^23,63^. About 62.5% of nitrate were removed at pH 2, while the results decreased to 48.3%, 43.9%, 35.0%, and 16.5% respectively at pH values of 4, 6, 8, and 10. The rapid decline in the nitrate removal rate is attributed to a rapid passivation of the iron surface under alkaline conditions, while the acidic condition assists in dissolving the surface (hydr)oxides layer, and reveals more active sites for the chemical reduction of nitrate^23^. Nevertheless, the denitration process exhibited a distinctive trend at a dosage of 6.0 g/L. Figure 6b shows that the nitrate removal rate was similar for different pH values, with the nitrate removal rate ranging from 94.3% to 96.7%. The minimal influence of the initial pH may be explained by the presence of sufficient contacts between nitrate and iron, although a high pH value is usually considered unfavorable for nitrate reduction because of the precipitation of iron (hydr)oxide.

For further comprehension of the relationship between the passivation process and pH, an index named “conversion efficiency” was introduced to explore the effectivity of nZVI in nZVI-Pd/NG. At a dosage of 6.0 g/L, the conversion efficiency of nZVI-Pd/NG ranged from 31.4% to 52.6% for all initial pH sets. When 2.0 g/L material was added to the system, the conversion efficiency at pH of 2 varied from 62.5%-100%, with the interval shifting to the lower end as the initial pH value increased. Only 16.5% to 26.4% conversion efficiency were achieved at an initial pH of 10. We inferred that a higher nZVI utilization rate was achievable in an acidic environment, while an excess dosage of nZVI-Pd/NG would ensure a high nitrate removal rate for a wide pH range.

**Figure 7:**
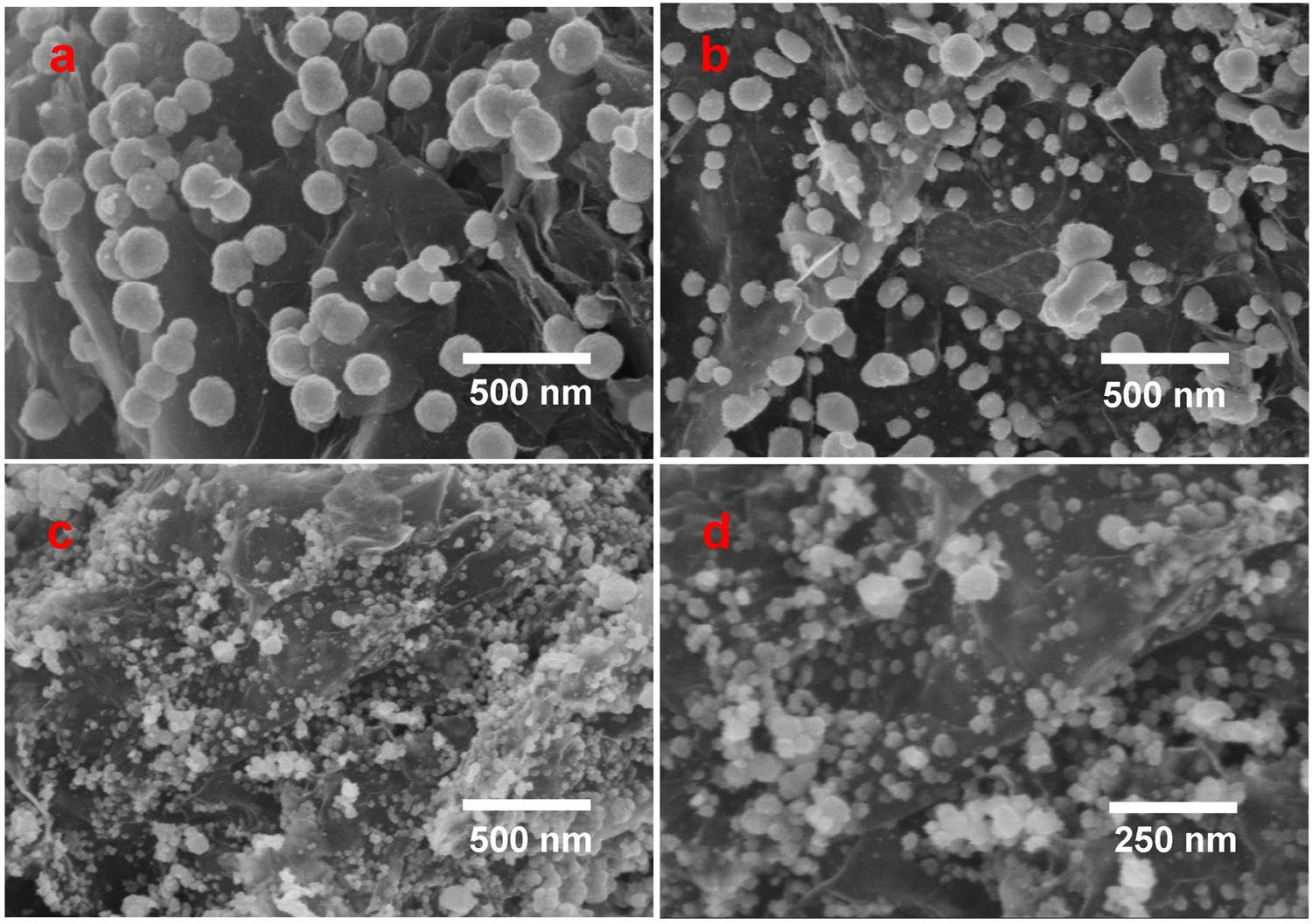
SEM micrographs of (**a**) raw nZVI-Pd/NG, (**b**) used nZVI-Pd/NG at 6.0 g/L dosage; (**c**,**d**) used nZVI-Pd/NG at 2.0 g/L dosage (initial nitrate concentration = 100 mg N/L, initial pH = 7, and temperature = 293 K).

Different degrees of nitrate reduction were achieved at different dosage levels, and the corresponding transformation of the material surface was further investigated by SEM analysis (Fig. 7). Compared with the freshly prepared nZVI-Pd/NG, nanoparticles on used nZVI-Pd/NG showed more irregular shapes and smaller particle sizes. Small agglomerates were present in used nZVI-Pd/NG at 6.0 g/L dosage, while nanoparticles were largely distributed separately on graphene surface. The used material at 2.0 g/L dosage showed particles of even smaller sizes (within 50 nm), while particles were detected on the surface and within the layer of graphene. The decrease in precipitated particle sizes suggest a higher utilization of reactive nZVI, consistent with the conversion efficiency results. Uniformly precipitated products on the surface of HCl-BC (supporting material) are proposed to slow down the passivation of nZVI, and help to maintain the reactivity of nZVI^64^. In our study, graphene functions similarly in offering a high surface area for the uniform precipitation of iron (hydr)oxides, so that passivation on the nZVI surface is weakened and nitrate is fully reduced. Moreover, the electron transfer between graphene and nZVI in composites also occurred, with the reaction center moving to the graphene surface, so that surface passivation of nZVI is retarded^22^.

## Conclusions

In the present study, a novel material, nZVI-Pd/NG, was synthesized using the wet impregnation method for denitration. It substantially improved the nitrate removal efficiency by complexation of nZVI on graphene with deposited Pd. The nZVI particles exhibited a uniform dispersion and smaller nano-sizes when supported on graphene. A nitrate removal rate of 97.0% was achieved, with a maximum reaction rate of 0.0531 min^−1^. The activation energy for the nitrate reduction by nZVI-Pd/NG was significantly lower than that of nZVI, highlighting the superior performance of the new composite. The catalytic effect of palladium was also elucidated, and a better selectivity for favorable end products was obtained. Graphene also exhibited the ability to lessen the passivation process of nZVI by offering enough sites for precipitation and enhancing the electron transfer during the reaction. Although nZVI-Pd/NG shows a great potential for nitrate reduction, further studies are required to examine the effects of other ions and improve the nitrogen selectivity.

## Methods

### Synthesis of nZVI-Pd/NG

The nZVI-Pd/NG introduced in this study was synthesized as follows: (1) About 4.830 g of FeCl_3_·6H_2_O and 0.277 g of PdCl_2_ were dissolved in DI water using an ultrasonic vibration for 1 h. (2) About 0.833 g of graphene was then added to the solution and continuously stirred with an electric blender for 3 h. (3) The mixture was transferred to a 1-L three-necked flask and purged with N_2_ for 30 min while stirring at 250 rpm. (4) A NaBH_4_ solution was prepared and added dropwise into the flask using a peristaltic pump, under continuous purging with N_2_ and stirring at 250 rpm. (5) The synthesized nZVI-Pd/NG was then separated magnetically, washed thrice using DI water, lyophilized for 24 h, and then grinded for use. It was a black powder with a mass ratio of nZVI: Pd: NG of 3:0.5:2.5. This ratio was determined based on the experimental data that showed an optimum nitrate removal efficiency (Fig S4). The chemical equations associated with the synthesis process are as follows:

3 $$2{{\rm{Pd}}}^{2+}+{{\rm{BH}}}_{4}^{-}+3{{\rm{H}}}_{2}{\rm{O}}\to 2{{\rm{Pd}}}^{0}+{{\rm{H}}}_{2}{{\rm{BO}}}_{3}^{-}+4{{\rm{H}}}^{+}+2{{\rm{H}}}_{2}$$

4 $$4{{\rm{Fe}}}^{3+}+3{{\rm{BH}}}_{4}^{-}+9{{\rm{H}}}_{2}{\rm{O}}\to 4{{\rm{Fe}}}^{0}+3{{\rm{H}}}_{2}{{\rm{BO}}}_{3}^{-}+12{{\rm{H}}}^{+}+6{{\rm{H}}}_{2}$$

### Synthesis of nZVI, nZVI-Pd, nZVI/NG, and Pd/NG

The synthetic methods for other materials were adjusted based on that for nZVI-Pd/NG. For nZVI, PdCl_2_ and graphene was removed, while for nZVI-Pd, graphene was removed, whereas for nZVI/NG, PdCl_2_ was removed; and for the Pd/NG, FeCl_3_·6H_2_O was eliminated. The NaBH_4_ concentration was slightly lowered based on the amount of metal prepared for reduction. Since the Pd/NG could not be magnetically separated, centrifugation was applied.

### Experimental procedure

The nitrate reduction tests were performed in a 50 mL headspace vial. Prior to each test, 100 mg N/L of nitrate was placed in the vial and bubbled with nitrogen gas. Before investigation of the impact of the initial pH, the solution’s pH was adjusted using a 1 M HCl/NaOH solution to the desired values (i.e., pH of 3, 5, 7, 9, and 11). The initial pH was unadjusted and considered as 7, when unspecified. The effect of the nZVI-Pd/NG dosage was investigated by applying dosage gradients of 2.0, 4.0, 6.0, 8.0, and 10.0 g/L to the system. The dosage of different materials (i.e., nZVI-Pd/NG, nZVI/NG, nZVI, nZVI-Pd and Pd/NG) differed based on the equivalent dosage of 3.0 g/L nZVI or 0.5 g/L Pd. After a specific dosage of a material was added to the system, the vial was further aerated with nitrogen gas before sealing. The experiments were conducted in an oscillator at a rotation rate of 250 rpm. The ambient temperature was 293 K, except during the investigation of the effect of temperature (temperatures of 283 K, 293 K, 303 K, and 313 K were employed). Samples were collected at intervals of 3, 5, 10, 15, 30, 60, 90, and 120 min, and the intervals were adjusted based on the test purpose. Samples were filtered using a 0.22 μm filter membrane and used for determining the concentrations of nitrogen species.

The nitrate removal rate was calculated from the following equation:

5 $${\rm{Nitrate}}\,{\rm{removal}}\,{\rm{rate}}=\frac{{[N{O}_{3}^{-}]}_{0}-{[N{O}_{3}^{-}]}_{t}}{{[N{O}_{3}^{-}]}_{0}}\times 100 \% $$

where ${[N{O}_{3}^{-}]}_{0}$ and ${[N{O}_{3}^{-}]}_{t}$ represent the initial and residual nitrate concentrations (mg N/L) at time t. The N_2_ selectivity was used to calculate the proportion of nitrogen in the reduction product according to the following equation (nitrite was negligible) given by:

6 $${{\rm{N}}}_{2}\,{\rm{selectivity}}=\frac{{[N{O}_{3}^{-}]}_{0}-{[N{O}_{3}^{-}]}_{t}-{[N{H}_{4}^{+}]}_{t}}{{[N{O}_{3}^{-}]}_{0}-{[N{O}_{3}^{-}]}_{t}}\times 100 \% $$

where ${[N{H}_{4}^{+}]}_{t}$ is the final ammonium concentration (mg N/L).

The total nitrogen (TN) at time t was calculated by the summation of nitrate and ammonia at the same time:

7 $${{\rm{TN}}}_{t}={[N{O}_{3}^{-}]}_{t}+{[N{H}_{4}^{+}]}_{t}$$

The conversion efficiency of the material was calculated according to the actual usage of nZVI as follows:

8 $${\rm{conversion}}\,{\rm{efficiency}}=\frac{{[N{O}_{3}^{-}]}_{0}-{[N{O}_{3}^{-}]}_{t}}{dosage\times \frac{1}{2}}\times \frac{56}{14000}\times (2.5 \sim 4)\times 100 \% $$

where dosage is the practical dosage (g/L) of nZVI-Pd/NG.

The kinetics for the nitrate reduction process was examined through pseudo-first kinetic model that was indicated as suitable for the nitrate reduction process by nZVI^16,19^ and expressed as:

9 $$\mathrm{ln}\,\frac{{[N{O}_{3}^{-}]}_{0}}{{[N{O}_{3}^{-}]}_{t}}={k}_{obs}t$$

where k_obs_ represents the observed rate constant of the pseudo first-order reaction (min^−1^).

The catalytic activity of the nitrate reduction process was deduced from the relationships between the temperature and rate constants, that is described by the Arrhenius equation^56^ as:

10 $$\begin{array}{c}\,K=A\times {e}^{-\frac{Ea}{RT}}\end{array}$$

where K refers to the measured first-order rate constant, E_a_ stands for the activation energy (kJ·mol^−1^), A is the Arrhenius frequency factor, R is the ideal gas constant (8.314 J/mol·K), and T is the absolute temperature (K). Integrating Eq. (10) produces Eq. (10) given as:

11 $$\begin{array}{c}lnK=-\frac{Ea}{R}\times \frac{1}{T}+lnA\end{array}$$

The activation energy E_a_ was determined by plotting K versus 1/T, with the slope yielding its value.

### Analytical methods

The concentrations of nitrate were analyzed using spectrophotometry with a UV–Vis spectrophotometer (METASH UV-8000) according to the absorbance peaks at 220 nm and 275 nm, while the concentrations of ammonia were measured by a HACH DR3900 spectrophotometer. The pH of various solutions were measured with a pH meter (HACH HQ11d). Scanning electron microscopy (SEM, Hitachi S4800) images and energy dispersive spectrometer (EDS) spectra were obtained to characterize the morphology and elemental content of various materials. The diameters of nZVI particles were measured by a Zetasizer Nano ZS 90 through dispersion of nZVI samples in ethanol by ultrasound.

The Brunauer-Emmett-Teller (BET) specific surface areas and BJH pore size distributions were determined by nitrogen adsorption and desorption at 77.3 K on a Micromeritics ASAP 2460 instrument. The samples were pretreated at 473 K under vacuum (1.33 Pa), and the isotherms were classified according to Brunauer-Deming-Deming-Teller (BDDT)^23^.

Powder X-ray diffraction (XRD) patterns were recorded on a Brucker D8 ADVANCE system using a Cu Kα X-ray radiation source at 45 kV and 200 mA. The samples were scanned from 10° to 90° at a scan speed of 2° min^−1^. The XRD patterns of the samples were analyzed by comparison with references in the Joint Committee on Powder Diffraction Standards (JCPDS) diffraction data files (JADE 6.5, Materials Data Inc.)^21^.

The X-ray photoelectron spectroscopy (XPS) analyses were performed using an ESCALAB 250Xi spectrometer (Thermo Fisher, USA) with Al Ka X-ray (1486.6 eV) to investigate the oxidation state of Fe and Pd^45^. The nZVI-Pd/NG samples after the reaction were collected and washed using DI water. The catalyst used was lyophilized for 24 h and maintained under anaerobic condition, and then carefully packed onto the XPS sampling templates.

## Supplementary information

Figure S1.

Figure S2.

Figure S3.

Supplementary information.
