# Supplementary figures and images for: Effect and mechanism of graphene structured palladized zero-valent iron nanocomposite (nZVI-Pd/NG) for water denitration
Source: Sci Rep. 2020 Jun 18;10:9931. doi: 10.1038/s41598-020-66725-z (PMC7303133; doi:10.1038/s41598-020-66725-z)

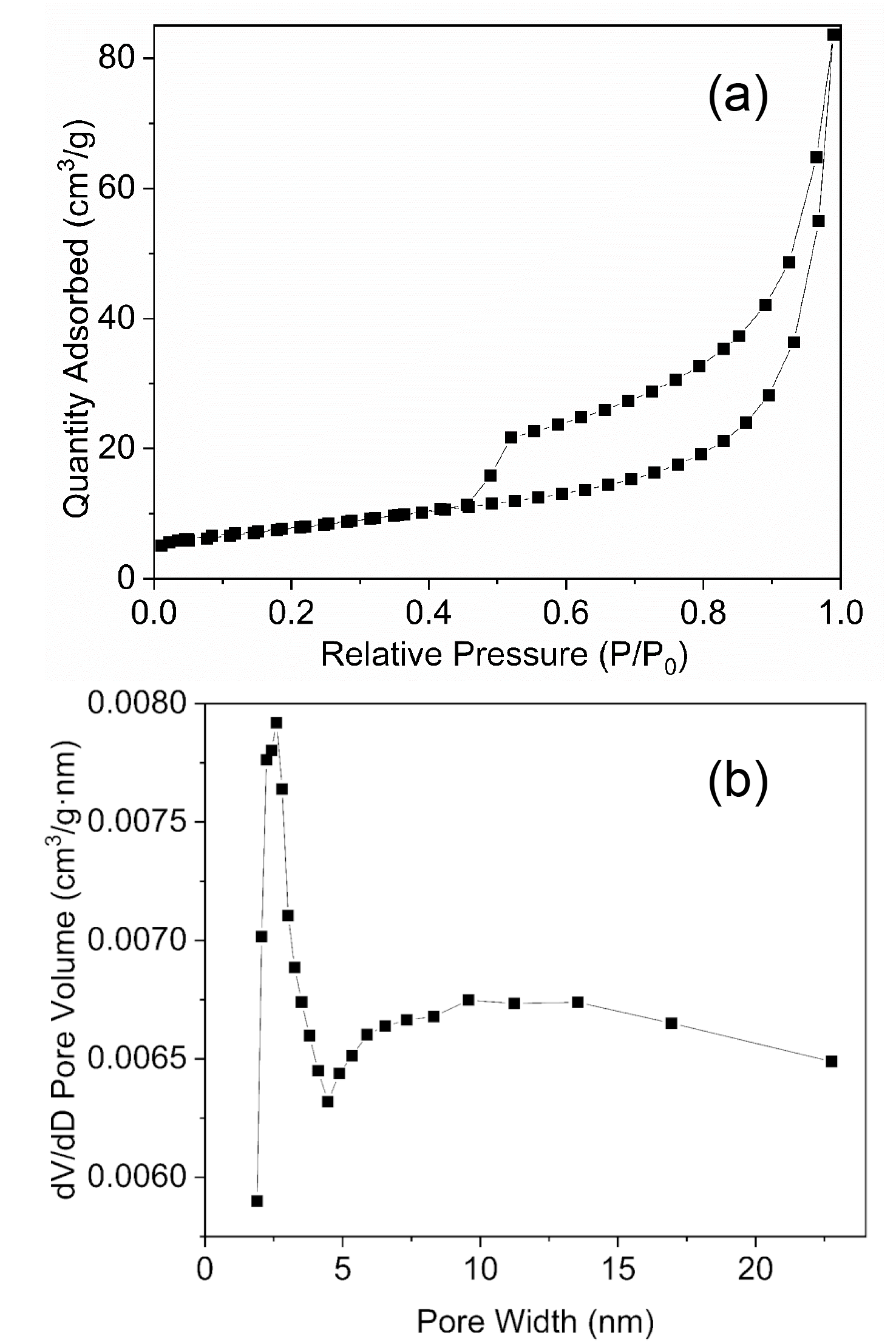

Supplement: Supplementary file 1 — Figure S1. [file 41598_2020_66725_MOESM1_ESM.tif]

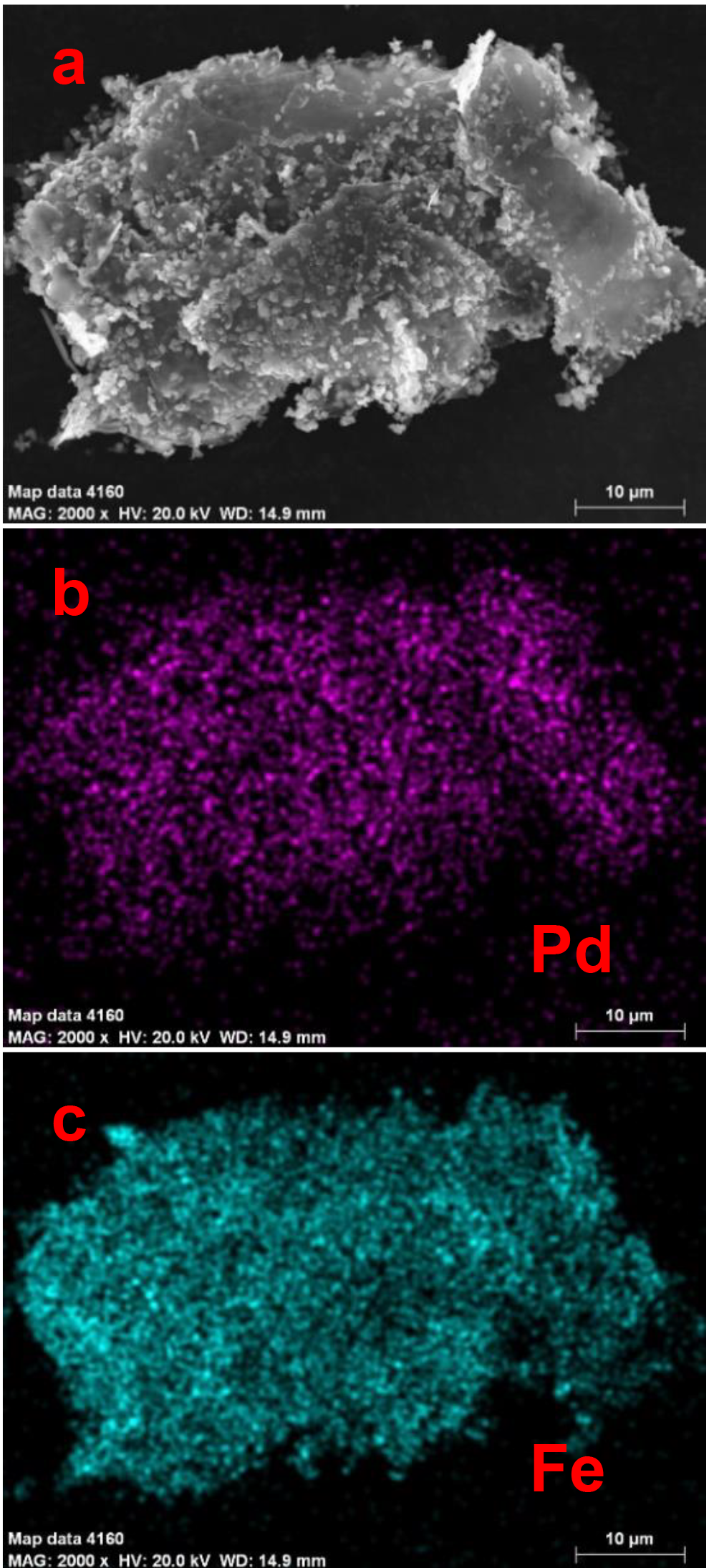

Supplement: Supplementary file 2 — Figure S2. [file 41598_2020_66725_MOESM2_ESM.tif]

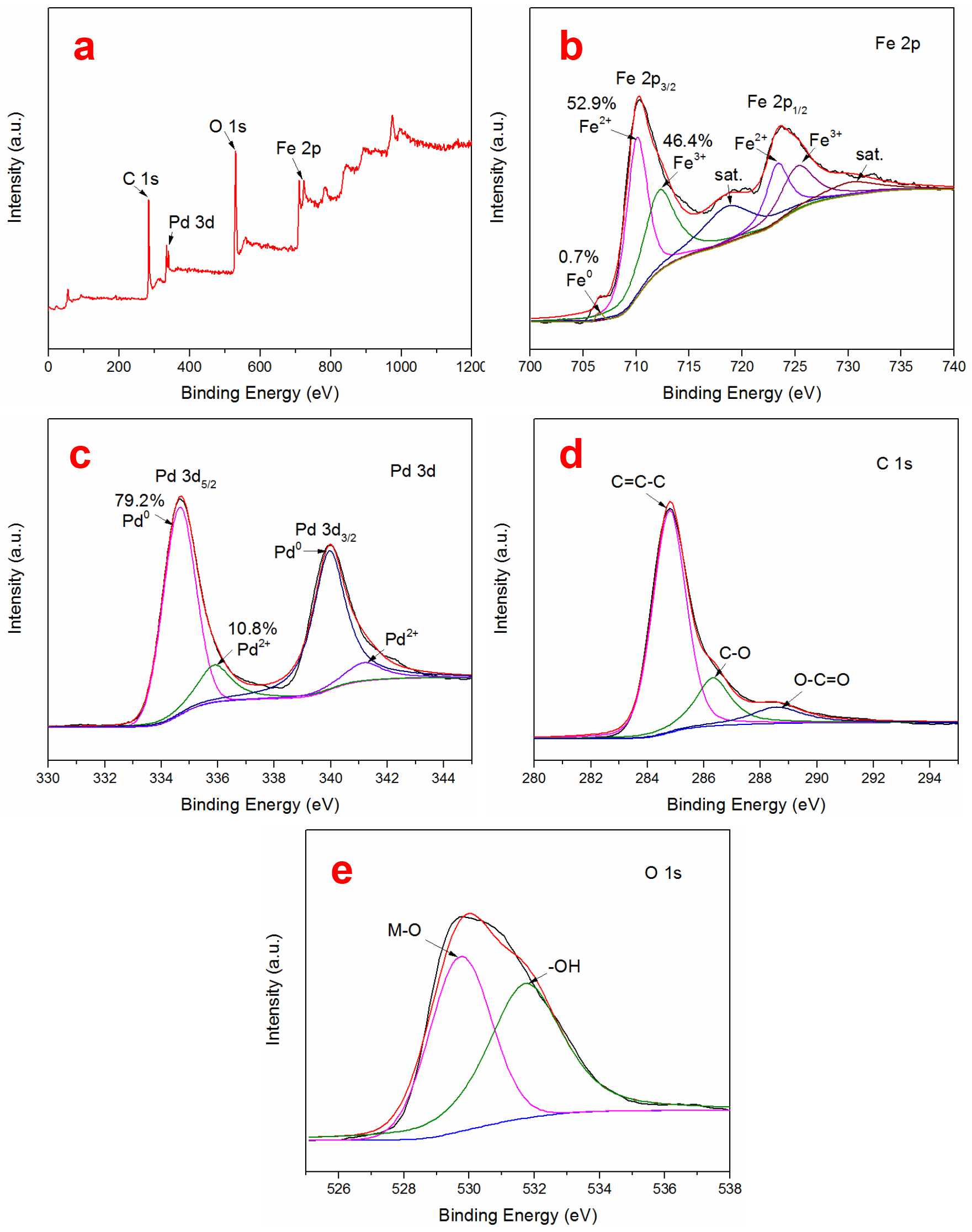

Supplement: Supplementary file 3 — Figure S3. [file 41598_2020_66725_MOESM3_ESM.tif]
