# Supplementary material for: Effect and mechanism of graphene structured palladized zero-valent iron nanocomposite (nZVI-Pd/NG) for water denitration
Source: Sci Rep. 2020 Jun 18;10:9931. doi: 10.1038/s41598-020-66725-z (PMC7303133; doi:10.1038/s41598-020-66725-z)
Supplement: Supplementary file 4 — Supplementary information. [file 41598_2020_66725_MOESM4_ESM.pdf]

Supplementary Information

Effect and mechanism of graphene structured palladized zero-valent iron nanocomposite (nZVI-Pd/NG) for water denitration

Xiangfeng Huang [a], *Feifan Zhang* [a], *Kaiming Peng* [a], *Jia Liu* [a], *Lijun Lu* [a], *Shiyang Li* [a*]

[a] College of Environmental Science and Engineering, State Key Laboratory of Pollution Control and Resource Reuse, Ministry of Education Key Laboratory of Yangtze River Water Environment, Shanghai Institute of Pollution Control and Ecological Security, Tongji University, Shanghai 200092, China

[*]Corresponding author:

Tel/Fax: +86 21 65982399. Email: lishiyang@tongji.edu.cn

This file contains the following information:

Table: 2

Figure: 4

List of Tables and Figures

Table S1 BET surface area, average pore size and pore volume of nZVI, graphene and nZVI-Pd/NG

Table S2 Reaction rate of nitrate reduction by nZVI and nZVI-Pd/NG at different temperature

Fig S1 (a) Adsorption isotherm and (b) pore width distribution of nZVI-Pd/NG

Fig S2 SEM, EDS images of fresh nZVI-Pd/NG. (a) SEM image of nZVI-Pd/NG; (b)-(c) EDS images of Pd, Fe elements

Fig S3 XPS spectra of (a) full survey of fresh nZVI-Pd/NG; (b-e) Fe, Pd, C, O of fresh nZVI-Pd/NG

Fig S4 (a) Nitrate removal rate of nZVI-Pd/NG with different nZVI: NG ratio; (b) SEM graphs of nZVI-Pd/NG with different nZVI: NG ratio

Table S1 BET surface area, average pore size and pore volume of nZVI, graphene and nZVI-Pd/NG

	S_{BET}(m^2/g)	Pore size (nm)	Pore Volume (cm^3/g)
nZVI	11.05	12.62	0.026
nZVI-Pd/NG	28.07	10.04	0.13
Graphene	227.04	5.62	0.52

Table S2 Reaction rate of nitrate reduction by nZVI and nZVI-Pd/NG at different temperature*

Temperature	nZVI		nZVI-Pd/NG	
	reaction rate (min^{-1})	R^2	reaction rate (min^{-1})	R^2
283 K	0.0087	0.967	0.0393	0.982
293 K	0.0123	0.945	0.0531	0.994
303 K	0.0296	0.994	0.0926	0.993
313 K	0.0874	0.963	0.1650	0.980

*initial nitrate concentration= 100 mg N/L, initial pH=7, nZVI dosage=3.0 g/L, nZVI-Pd/NG dosage= 6.0 g/L

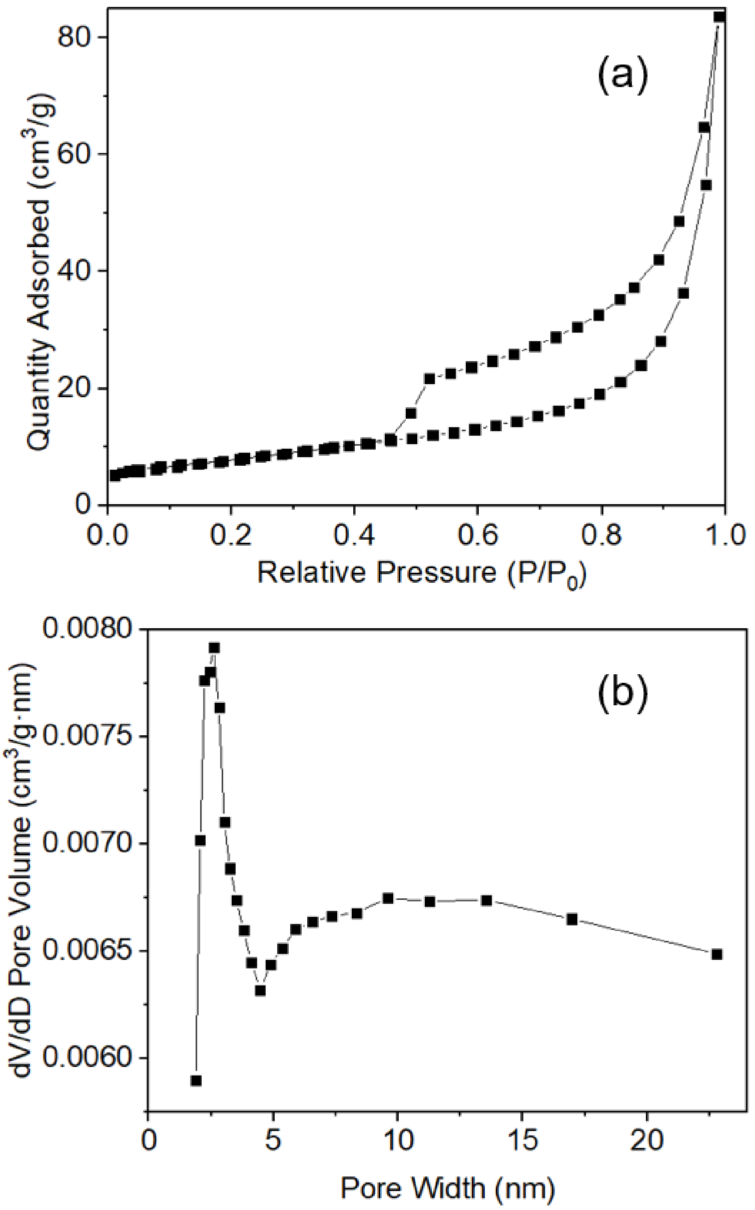

Fig S1 (a) Adsorption isotherm and (b) pore width distribution of nZVI-Pd/NG

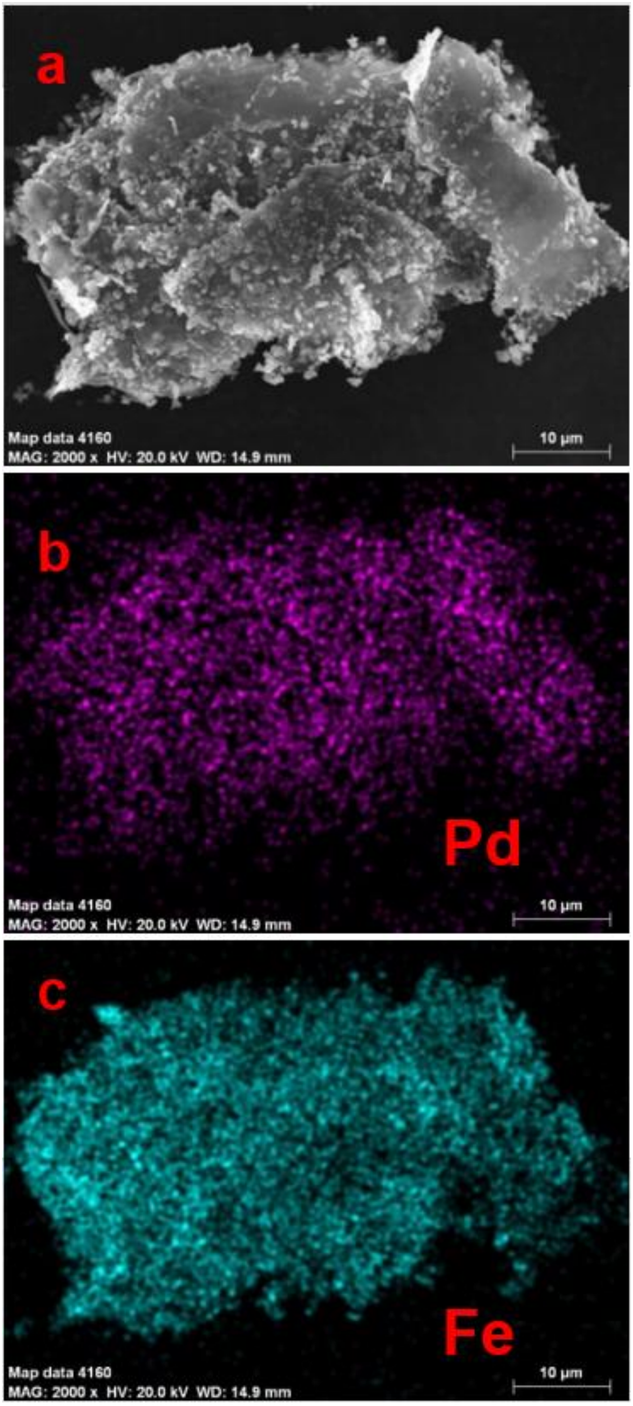

Figure S2 SEM, EDS images of fresh nZVI-Pd/NG. (a) SEM image of nZVI-Pd/NG; (b)-(c) EDS images of Pd, Fe elements.

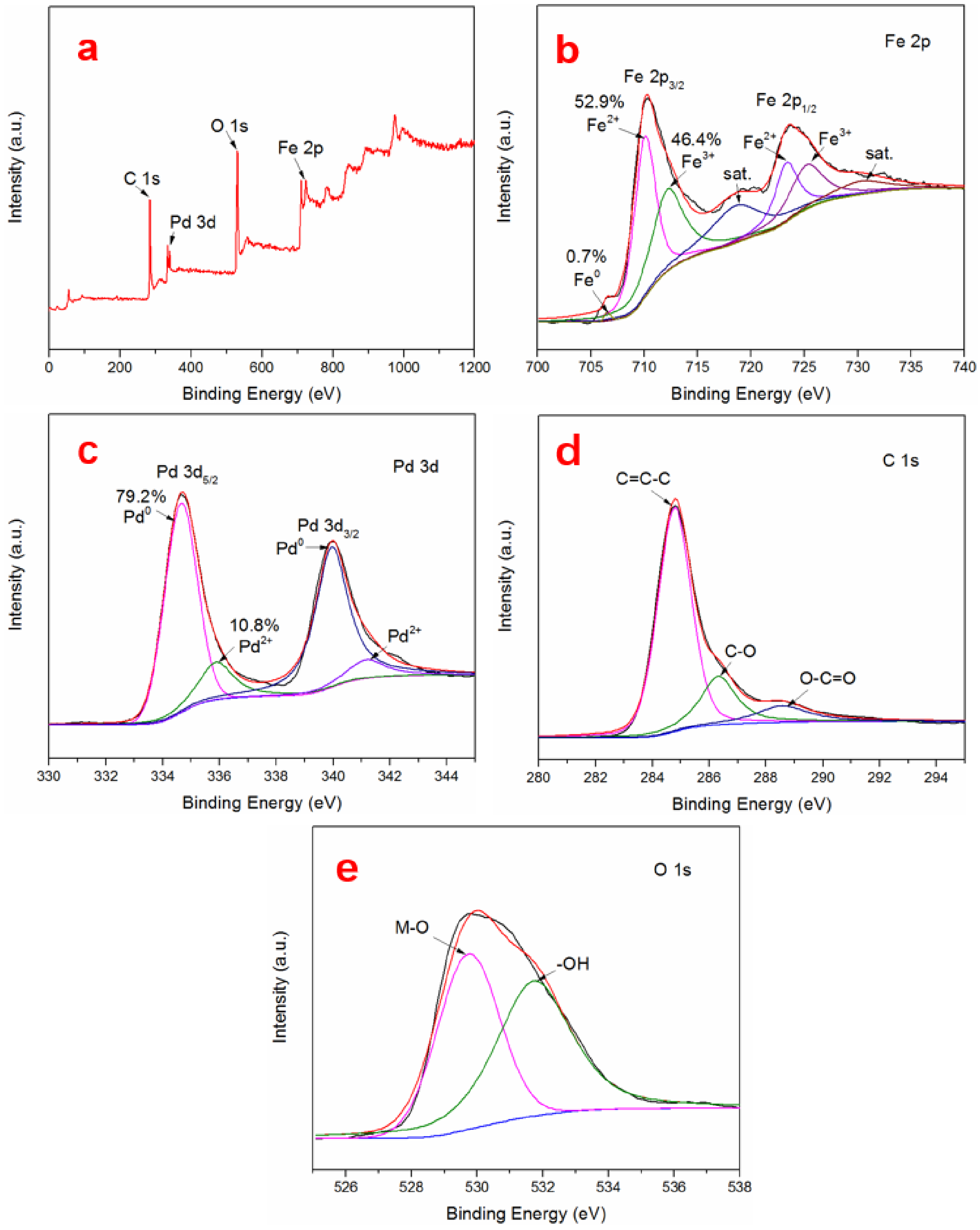

Figure S3 XPS spectra of (a) full survey of fresh nZVI-Pd/NG; (b-e) Fe, Pd, C, O of fresh nZVI-Pd/NG

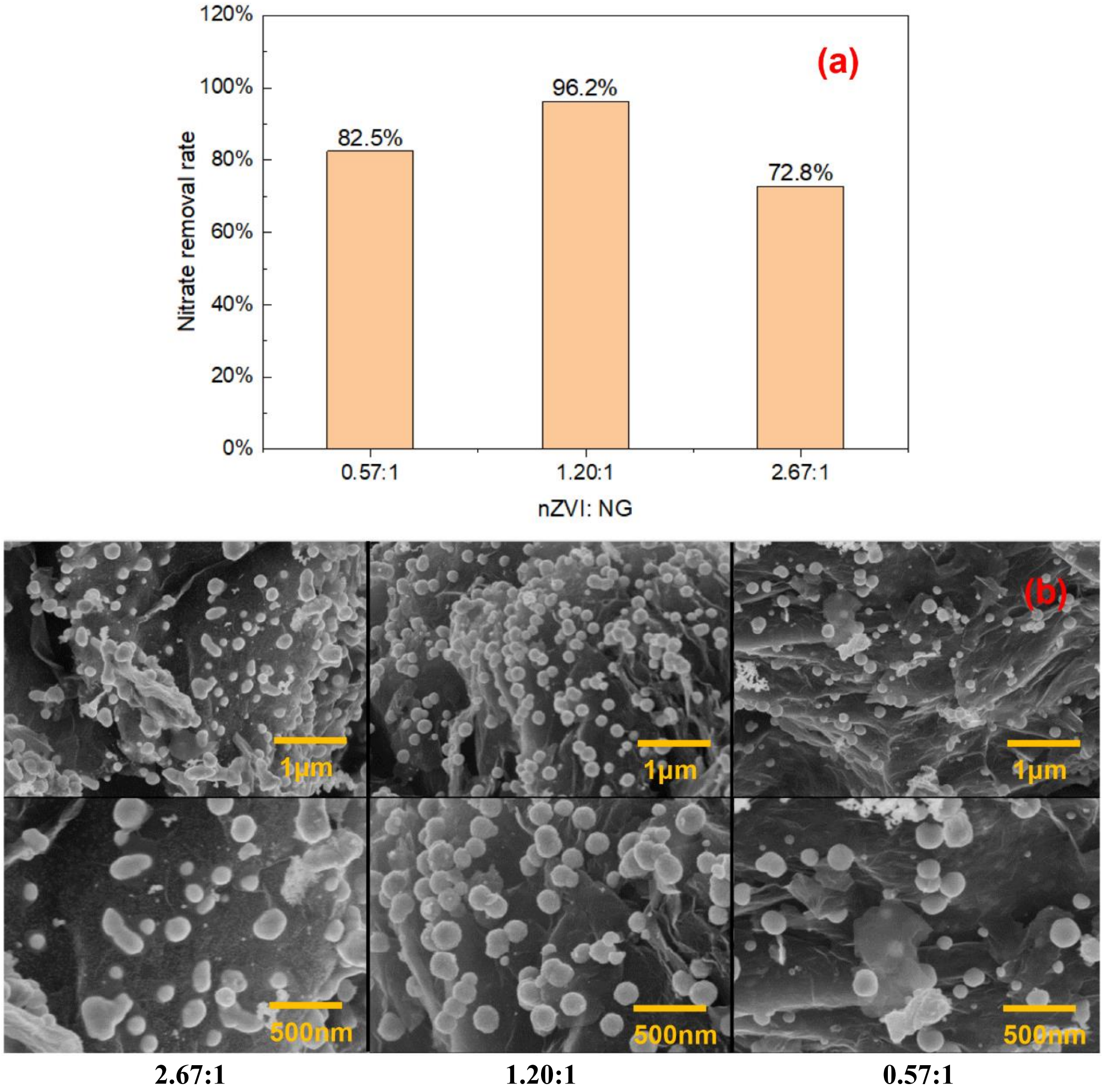

Figure S4 (a) Nitrate removal rate of nZVI-Pd/NG with different nZVI: NG ratio; (b) SEM graphs of nZVI-Pd/NG with different nZVI: NG ratio
